# Effect of Administration of an Equal Dose of Selected Dietary Chemicals on Nrf2 Nuclear Translocation in the Mouse Liver

**DOI:** 10.1155/2023/9291417

**Published:** 2023-04-10

**Authors:** Nadia Salem Alrawaiq, Ahmed Atia, Azman Abdullah

**Affiliations:** ^1^Department of Pharmacology, Faculty of Medicine, Universiti Kebangsaan Malaysia, Jalan Yaacob Latif, 56000 Cheras, Kuala Lumpur, Malaysia; ^2^Department of Pharmacology, Faculty of Pharmacy, Sebha University, Sebha, Libya; ^3^Department of Anaesthesia and Intensive Care, Faculty of Medical Technology, Tripoli University, Tripoli, Libya

## Abstract

Certain dietary chemicals influenced the expression of chemopreventive genes through the Nrf2-Keap1 pathway. However, the difference in Nrf2 activation potency of these chemicals is not well studied. This study is aimed at determining the difference in the potency of liver Nrf2 nuclear translocation induced by the administration of equal doses of selected dietary chemicals in mice. Male ICR white mice were administered 50 mg/kg of sulforaphane, quercetin, curcumin, butylated hydroxyanisole, and indole-3-carbinol for 14 days. On day 15, the animals were sacrificed, and their livers were isolated. Liver nuclear extracts were prepared, and Nrf2 nuclear translocation was detected through Western blotting. To determine the implication of the Nrf2 nuclear translocation on the expression levels of several Nrf2-regulated genes, liver RNA was extracted for qPCR assay. Equal doses of sulforaphane, quercetin, curcumin, butylated hydroxyanisole, and indole-3-carbinol significantly induced the nuclear translocation of Nrf2 with different intensities and subsequently increased the expression of Nrf2-regulated genes with an almost similar pattern as the Nrf2 nuclear translocation intensities (sulforaphane > butylated hydroxyanisole = indole-3-carbinol > curcumin > quercetin). In conclusion, sulforaphane is the most potent dietary chemical that induces the Nrf2 translocation into the nuclear fraction in the mouse liver.

## 1. Introduction

Aerobic organisms are constantly producing reactive oxygen species (ROS), such as superoxide, hydrogen peroxide, and hydroxyl free radicals, as a natural by-product of oxygen metabolism. Free radicals are molecules with one or more unpaired electrons. Free radicals cause damage when they react with other molecules to find electrons to pair with their unpaired electrons. The other molecules then lost their electrons, causing them to become free radicals themselves, thus creating a chemical chain reaction of free radical production [1]. Oxidative stress is a condition that occurs when there is an imbalance between the production and accumulation of free radicals (reactive oxygen species) in the living system and the ability of the living system to detoxify these reactive products [2]. Oxidative stress in the body due to pollutants, xenobiotics, and certain foods can expedite the formation of free radicals. The free radical chain reaction may cause damage to cellular homeostasis due to its potential in causing alterations in the lipid, protein, and DNA structure. The damaged molecules may initiate mutation and growth of tumours [1]. Reactive oxygen species (ROS) play an essential role in cell signaling and homeostasis. However, high levels of ROS are responsible for various medical conditions, including ageing, cancer, inflammation, and chronic illnesses [1]. Therefore, exogenous antioxidants from the chemicals in foods have been used to prevent or repair the oxidative damage caused by ROS [3]. Another way to protect against oxidative damage is perhaps to increase the production of endogenous antioxidant/phase II detoxifying enzymes via the activation of the antioxidant signaling route [4].

Several dietary compounds are recognized as potential agents that interfere with disease processes. Certain vegetables and fruits, such as broccoli, blueberries, and cacao beans, are known to be especially highly protective as they contain excessive amounts of phytochemicals such as isothiocyanates, polyphenols, and flavonoids [5]. Upon entry to cells, these phytochemicals can directly hunt down free radicals and can also activate electrophilic stress signals that stimulate various cellular signaling pathways, such as the Keap1-Nrf2/ARE pathway which induces the expression of cytoprotective phase II proteins [4]. The nuclear erythroid 2-related factor 2 (Nrf2) is a transcription factor that protects against oxidative stress [6]. The Keap1-Nrf2 signaling pathway comprises the Kelch-like ECH-associating protein 1 (Keap1), Nrf2, and the antioxidant responsive element (ARE) cis-acting element. This pathway serves as a master oxidation-reduction system, which protects cells from injury and death [7].

In the absence of stress, Keap1 is an E3 ubiquitin ligase substrate adaptor that targets Nrf2 for rapid proteasomal degradation, which results in a limited cytoplasmic concentration of Nrf2 [8]. When exposed to electrophiles or oxidative stress, several highly reactive cysteine residues of Keap1 are modified to prevent it from targeting Nrf2 for proteasome degradation, resulting in rapid accumulation of Nrf2 proteins, which translocate into the nucleus and undergo dimerization with small musculoaponeurotic fibrosarcoma (sMaf) proteins, which are then able to target genes coding for a wide range of cytoprotective enzymes, such as antioxidant enzymes, that have ARE (antioxidant response elements) elements in their regulatory regions [9]. Intracellular endogenous antioxidant genes, such as NAD(P)H: quinone oxidoreductase-1 (NQO1), superoxide dismutase (SOD), heme oxygenase-1 (HO-1), glutathione s-transferases (GST), and *γ*-glutamylcysteine synthetase (*γ*-GCS) also called glutamate cysteine ligase (GCL), catalase(CAT), etc., are increased to maintain homeostasis [10].

Nrf2 induced the expression of many detoxification phase II enzyme genes such as glutamate cysteine ligase catalytic subunit (GCLC), catalase (CAT), and glutathione peroxidase-1 (GPX1), which are reported in this study, and the function of these phase II enzymes is mainly to metabolize harmful carcinogens to inert metabolites, which could prevent the formation of cancer. This concept is known as cancer chemoprevention [11]. The expression of many phase II enzymes is regulated by the transcription factor Nrf2 [12]. Phase II enzymes are important for cellular defence by enhancing the removal of toxic metabolites and free radicals, thereby playing a possible protective role against the development of cancer and other diseases, which is an integral component of cancer chemoprevention [11]. The redox-sensitive transcription factor Nrf2 mediates cellular defence against chemical and oxidative stress. Oxidative, chemical, and electrophilic stresses lead to Nrf2 activation and nuclear translocation, which in turn upregulates the expression of several antioxidant defence genes and various phase II detoxifying enzymes, which detoxifies any potential carcinogen thus preventing the formation of cancer [11, 13, 14]. Therefore, in healthy mice (and most possibly in healthy individuals), activation of the Nrf2 pathway by administration of dietary supplements would upregulate the expression of antioxidant proteins and phase II detoxifying enzymes, thus potentially hindering any opportunity for cancer initiation.

Phytochemicals such as sulforaphane (SUL), curcumin (CUR), quercetin (QRC), and indole 3 carbinol (I3C) have been shown to activate the Nrf2 pathway in the livers of animals such as rats and mice [15–20] (Table 1). We have previously shown that the treatment of mice with equal dose (50 mg/kg body weight) of SUL, CUR, QRC, I3C, and BHA for 14 days resulted in different patterns of expression of phase II enzymes regulated by Nrf2 such as NQO1 and HO-1 in the liver [21, 22]. At a dose of 50 mg/kg body weight for 14 days, administration of SUL resulted in the highest NQO1 expressional level in the liver, followed by I3C and BHA (equal expression level), QRC, and CUR [21]. Also, at the same dose of 50 mg/kg, SUL administration resulted in the highest level of expression of HO-1 in the mouse liver, followed by I3C and BHA (equal expression level), CUR, and QRC (lowest expression) [22].

Sulforaphane is a potent Nrf2 inducer that could increase the expression of many cytoprotective enzymes. The central carbon of its electrophilic isothiocyanate group (−N=C=S) reacts with nitrogen-, sulfur-, and oxygen-based nucleophiles [23–25]. It is postulated that the central carbon of the isothiocyanate (−N=C=S) group of sulforaphane is highly electrophilic and reacts avidly with sulfhydryl groups. The highly electrophilic nature of the central carbon atom of the isothiocyanate (–N=C=S) group is likely to induce Nrf2 transcriptional activity through its modification of specific Keap1 cysteine thiols [24–26].

Curcumin exerts both direct and indirect antioxidant effects by scavenging ROS and inducing the expression of cytoprotective proteins in an Nrf2-dependent way [20]. ROS scavenging capacity of curcumin (direct antioxidant effect) is mainly attributed to its structure as a bis-*α*,*β*-unsaturated-diketone of the two ferulic acid units, connected through a methylene group. The direct scavenging of radicals and other reactive oxygen species is due to the presence of two phenolic functional groups and the *β*-diketo moiety in curcumin, which is known as the Michael acceptor group [27].

Quercetin is an example of a dietary chemical that requires electrophilic conversion for inducer activity. ROS (e.g., O_2_^·^−) and reactive nitrogen species (RNS) (e.g., NO and ONOO−) are the primary targets of quercetin [28]. The presence of two pharmacophores within the quercetin molecule (characterized by the optimal configuration for free radical scavenging, i.e., the catechol group in the B ring and the OH group at position 3) configures quercetin antioxidant capacity. Quercetin is considered a weak electrophile due to the electron-donating effect of the 3-hydroxyl group and both aromatic rings. Quercetin is oxidized to a quinone when serving as an antioxidant, and such quinones react with thiols [29]. Quercetin is oxidized in the body to become a more electrophilic quinone methide [30]. Quinone methide is much more reactive toward thiolates. Therefore, researchers postulated that free radical-oxidized quercetin reacts with thiols in Keap1, thus causing Nrf2 nuclear translocation and transcriptional regulation of antioxidant-responsive genes.

I3C and its metabolite 3,3′-diindolylmethane (DIM) cause Nrf2 induction and many other cancer chemopreventive effects [18]. DIM, a major metabolite of I3C, is primarily responsible for the chemopreventive activity of I3C [18]. Interestingly, DIM, but not I3C, was detected in human plasma samples following supplementation with I3C [31]. DIM was found to be more effective in inducing the Nrf2-Keap1 activity compared to I3C.

Commonly added as an additive to preserve oils and fats, butylated hydroxyanisole (BHA) is a synthetic phenolic antioxidant. BHA is widely used as an antioxidant and preservative in food, food packaging, and medicines (Table 1). It is subjected to O-dealkylation by cytochrome P450 isozymes in the liver to produce tert-butylhydroquinone (TBHQ) which further breaks down into tert-butylquinone (TBQ), which are prooxidants [32, 33]. The chemopreventive effect of BHA is mediated mainly by its reactive metabolites THBQ and TBQ. Its chemopreventive properties are attributed to its ability to activate Nrf2, which regulates the bodily processes of detoxification and protection against oxidative stress [34].

The transcription factor nuclear factor-*κ*B (NF-*κ*B) is responsible for the expression of various genes involved in multitude cellular processes such as apoptosis, cell cycle, angiogenesis, and metastasis. A large number of NF-*κ*B target genes are involved in innate immune response, inflammation, and cancer, which includes cytokines, chemokines, proteases, NOS2, and COX2 [25]. In conditions related to oxidative stress, the presence of ROS can both activate and repress the NF-*κ*B signaling in a phase- and context-dependent manner. The NF-*κ*B pathway can have both anti- and prooxidant roles in the setting of oxidative stress [35].

Previous cellular-based and animal studies have indicated that SUL, CUR, QRC, I3C, and BHA are involved in the chemoprevention of liver malignancy [24, 34, 36–39]. It was also evident from the results of our lab that the administration of equal doses of different chemicals to mice resulted in different patterns of expressions of certain phase II enzymes regulated by Nrf2 in the liver. However, the reasons behind the difference in expressions are not clear. Therefore, this study is aimed at investigating whether the difference in phase II enzymes expression pattern is due to the difference in Nrf2 translocation into the nuclear fraction of the liver, despite the same dose of chemicals being administered to the animals [21, 22]. It would also be pertinent to investigate whether the different patterns of expression of phase II enzymes are influenced by the difference in the intensity of Nrf2 expression levels in the nuclear fractions of the liver induced by equal doses of dietary chemicals with different structural properties.

In most tissues, cells are exposed to frequent changes in levels of oxidative stress and inflammation. Oxidative stress and inflammation can trigger a multitude of cellular response through a variety of signaling pathways [40]. Nrf2 and NF-*κ*B are the two key transcription factors that regulate cellular response to oxidative stress and inflammation, respectively. Pharmacological and genetic studies suggest that there is functional cross-talk between these two important pathways [41]. The deficiency of Nrf2 elevates the expression of NF-*κ*B, leading to increased production of inflammatory factors [42]. Additionally, NF-*κ*B could influence the expression of downstream target genes by regulating the transcription and activity of Nrf2. A large number of hepatoprotective reagents have been shown to activate the Nrf2-related gene expression and suppressed NF-*κ*B expression [41]. Some phase II antioxidant genes possess binding sites for NF-*κ*B in their regulatory element region [43–45]. With regard to these aspects, apart from Nrf2 nuclear translocation, we also investigated whether NF-*κ*B is translocated into the liver nuclear fraction and affect the expression of our genes of interest.

## 2. Materials and Methods

### 2.1. Chemicals and Materials

Butylated hydroxyanisole (BHA), indole-3-carbinol (I3C), quercetin (QRC), curcumin (CUR), Tween 20, dimethylsulfoxide (DMSO), and sodium chloride were purchased from Sigma-Aldrich (Seelze, Germany). Sulforaphane (SUL) was purchased from Santa Cruz Biotechnology (Santa Cruz County, California, USA). TRIzol reagent was manufactured by Life Technologies (Carlsbad, California, USA). iScript cDNA synthesis kit, iQ SYBR Green supermix (2×) kit, and MJ Mini thermal cycler were purchased from Bio-Rad (Hercules, California, USA). Real-time PCR primers were synthesized by Vivantis Technologies (Oceanside, California, USA). Chemiluminescence Western blotting detection reagents were bought from Amersham (Uppsala, Sweden). Nitrocellulose membrane and Ponceau red dye absolute solution were purchased from Sigma-Aldrich (Seelze, Germany). Primary polyclonal rabbit anti-mouse *β*-actin antibody was purchased from Abcam Biotech (Cambridge, UK). Primary polyclonal rabbit anti-mouse Nrf2, primary polyclonal rabbit anti-mouse NF-*κ*B-p50, primary polyclonal rabbit anti-mouse histone H3 antibodies, and RIPA lysis buffer were purchased from Santa Cruz Biotechnology (Dallas, Texas, USA). Anti-rabbit IgG peroxidase secondary antibody was purchased from Santa Cruz Biotechnology (Dallas, Texas, USA). All other chemicals were manufactured by Sigma-Aldrich unless otherwise stated.

### 2.2. Experimental Animals

Experimental animals used in this study were adult male ICR white mice obtained from the Universiti Kebangsaan Malaysia (UKM) Laboratory Animal Research Unit (LARU). Forty-two eight-week-old adult ICR male white mice (25-30 g) were used. Male mice were chosen in this study because their livers are larger than female mice. Furthermore, male mice are not subjected to oestrous cycles that can complicate the pharmacological studies of rodents. Most animal pharmacology/toxicology studies have been performed with male mice due to the fact that female mice undergo oestrous cycles that could create a tremendous amount of variability in experimental results [22]. The animals were treated after a period acclimatization of seven days at room temperature and relative humidity of 28.5°C and 50%, respectively. Mice were kept in conventional mouse cages at room temperature with a 12-hour light-dark cycle and fed with a standard pelletized diet (Gold Coin, Selangor, Malaysia) with tap water given ad libitum. The composition of the standard pelletized diet is given in Table 2. The standard pelletized food has been shown to be suitable for the growth of mice [46, 47]. The health of the animals was monitored daily by observing their behaviours and by measuring their body weight. Animals were maintained and handled according to the recommendations from the UKM Animal Ethics Committee (UKMAEC) which had approved the study design of the experiment (Approval Code: FP/FAR/2012/AZMAN/23-MAY/442-JUNE-2012-JUNE-2015).

### 2.3. Study Design

Forty-two male adult ICR white mice were divided into 7 groups (vehicle 1 control, vehicle 2 control, SUL, CUR, QRC, BHA, and I3C; *n* = 6 for each group). All chemicals were administered intraperitoneally at a dose of 50 mg/kg body weight for 14 days. Vehicle 1 (DMSO, Tween 20, and normal saline in the ratio of 0.05 : 0.1 : 0.85) was used to dissolve sulforaphane (SUL), curcumin (CUR), and quercetin (QRC) and was administered alone to the vehicle control 1 mouse group. Vehicle 2 (corn oil) which was used to dissolve butylated hydroxyanisole (BHA) and indole-3-carbinol (I3C) was administered alone to the vehicle control 2 mouse group. The chemicals were administered intraperitoneally (i.p.) at a dose of 50 mg/kg body weight for 14 consecutive days. In many previous studies (cited in Reference [22]), the dose of 50 mg/kg of several phytochemicals (SFN, indole-3-carbinol, quercetin, and curcumin) was effective in inducing several important antioxidant enzyme expressions. In a number of studies [21, 22, 48–50], two or more phytochemicals were given at a dose of 50 mg/kg in order to compare which phytochemical is more potent in producing positive health-protective effects. Therefore, the dose of 50 mg/kg was chosen in this current study because we wanted to observe which phytochemical is the most potent in inducing Nrf2 translocation and subsequently Nrf2-regulated gene expression, at that particular dose. The other reason we chose to give the dietary chemicals in terms of mg/kg body weight is to mimic the drug dosage given to humans, which is usually in terms of mg/kg. The animals were sacrificed by cervical dislocation once the 14-day treatment period was completed. The mice were not subjected to fasting before being sacrificed. Their livers were subsequently isolated, cleaned, snapped frozen in liquid nitrogen, and stored at -70°C until further use.

### 2.4. Nuclear Extract Preparation

Nuclear extracts from mouse liver tissues were prepared according to a procedure previously described by Atia et al. with slight adjustments [13]. Briefly, 100 mg of liver tissue was homogenized in 1 ml of cold buffer A [50 mM NaCl, 10 mM Hepes pH 8, 1 mM EDTA, 0.5 mM sucrose, 0.5 mM spermidine, 0.15 mM spermine, and 0.2% Triton X-100 (*v*/*v*)] at 4°C. The resulting homogenate was incubated on ice for 10 min and was subsequently subjected to centrifugation at 2500 *g* for 5 min at 4°C. The resulting supernatant (cytosolic fraction) was transferred into new microcentrifuge tubes and kept in -80°C until further use. The pellet was suspended in 0.5 ml of buffer B [2.5 mM NaCl, 0.5 mM Hepes–KOH, 0.05 mM EDTA, 0.025 mM spermidine, 0.075 mM spermine, and 25% glycerol] followed by centrifugation for 3 min at 1200 *g* at 4°C. The supernatant was discarded, and the pellet was resuspended in 50 *μ*l of cold buffer C [17.5 mM NaCl, 0.5 mM Hepes–KOH, 0.05 mM EDTA, 0.025 mM spermidine, 0.075 mM spermine, 25% glycerol, 0.4 mM PMSF, 4.5 *μ*l protease inhibitor cocktail in DMSO, and 0.6 mM 2-mercaptoethanol]. The suspension was then incubated on ice for 30 min and was subsequently centrifuged for 5 min at 1200 *g* at 4°C. The resulting supernatant (nuclear extract) was transferred into new microcentrifuge tubes and kept in storage at −80°C until further use.

### 2.5. Determination of Nrf2 Nuclear Translocation by Western Blot Analysis

Hepatic Nrf2 nuclear translocation and the expression levels of other proteins of interest in the nuclear and cytoplasmic fractions of the liver were determined using a previously described immunoblotting method [51]. Briefly, 100 *μ*g of liver protein (either from the nuclear or cytosolic fractions) was separated using 15% sodium dodecyl sulfate polyacrylamide gel (SDS-PAGE) electrophoresis. The proteins in the gel were then electrophoretically transferred to a nitrocellulose membrane. The membrane was then blocked at room temperature for 30 min using nonfat milk blocking solution. After blocking, the membrane was incubated with a primary antibody. The primary antibodies used in this experiment were those that recognized Nrf2, NF-*κ*B, and histone H3. After several washing steps, the membrane was incubated for 1 hour with a suitable secondary antibody. After another series of washing steps, the membrane was incubated with a detection solution. Protein bands were visualized using the enhanced chemiluminescence method. The intensity of the protein bands was quantified, relative to the signals obtained for housekeeping protein (histone H3 for the nuclear fraction and *β*-actin for the cytoplasmic fraction), using ImageJ software.

### 2.6. RNA Extraction

For the determination of gene expression levels, total RNA was isolated from liver tissues using TRIzol reagent, according to the manufacturer's instructions. Isopropyl alcohol (Sigma, USA) was added during each extraction step to precipitate the total RNA. The extracted total RNA pellet was then washed with 75% ethanol and dried before being dissolved in RNase-free water. Total RNA was stored at -80°C immediately after isolation. The concentration and purity of the extracted RNA were determined by NanoDrop spectrophotometer 2000c (Thermo Scientific, USA) at a wavelength of 260 nm (OD260). RNA with RNA integrity number (RIN) ranging from 7 to 10 and absorbance ratio of A260 to A280 ranging from 1.5 to 2.0 was used for cDNA synthesis.

### 2.7. Reverse Transcription

cDNA synthesis was performed using the iScript cDNA synthesis kit according to the manufacturer's instructions. Briefly, a volume (containing 1 *μ*g) of total RNA from each sample was added to a mixture of 4 *μ*l of 5× iScript reaction mix, 1 *μ*l of iScript reverse transcriptase, and a suitable volume of nuclease-free water (the final reaction mix volume is 20 *μ*l). The final reaction mixture was kept at 25°C for 5 min and 42°C for 30 min and heated to 85°C for 5 min in a thermocycler (TC-412, Techne, Barloworld Scientific, UK). The cDNA was then used as a template for amplification by polymerase chain reaction (PCR).

### 2.8. Quantification of Glutamate Cysteine Ligase Catalytic Subunit (GCLC), Catalase (CAT), and Glutathione Peroxidase-1 (GPX1) Gene Expression by Quantitative Real-Time PCR (qPCR)

Quantitative real-time PCR was performed on the MiniOpticon cycler (Bio-Rad, USA). The total reaction volume used was 20 *μ*l, consisting of 1 *μ*l of 10 *μ*M forward primer and 1 *μ*l of 10 *μ*M reverse primer (500 nM final concentration of each primer), 10.0 *μ*l of iQ™ SYBRTM Green Supermix (2×) (Bio-Rad, USA), 6.0 *μ*l of nuclease-free water, and 2.0 *μ*l of cDNA. Both forward and reverse primers for the genes of interest in this study were designed according to previous studies and synthesized by Vivantis Technologies (Oceanside, CA, USA). The primer sequences for our genes of interest are shown in Table 3. The thermocycling conditions were initiated at 95°C for 30 sec, followed by 40 PCR cycles of denaturation at 95°C for 15 sec and annealing/extension at 60°C for 30 sec. At the end of each cycle, a melting curve (dissociation stage) analysis was performed to determine the specificity of the primers and the purity of the final PCR product. All measurements were performed in triplicate, and no-template controls (NTC) were incorporated into the same set of PCR tubes to test for contamination by any assay reagents. Threshold cycles were determined for each gene, and quantification of templates was performed according to the relative standard curve method. The relative gene expression (DDCt) technique was used to analyze the real-time PCR data [52]. In short, the expression level of each target gene was given as a relative amount against GAPDH standard controls.

### 2.9. Statistical Analysis

Data are presented as the mean ± standard error of the mean (SEM). All values to be compared were analyzed for normality using the Shapiro-Wilk test. The Mann–Whitney test was used where nonparametric analysis was indicated. Statistical analysis was conducted using the SPSS software version 22. Results were considered statistically significant when *P* values were less than 0.05 (*P* < 0.05).

## 3. Results

### 3.1. Body Weight and Food Intake of Mice

The body weight and food intake of mice in each group were recorded every two days in order to investigate whether equal doses (50 mg/kg) of SUL, BHA, I3C, CUR, and QRC treatment affect the body weight gain and food intake in the control and treated mice. In the control and 50 mg/kg SUL-, BHA-, I3C-, CUR-, and QRC-treated mice, the body weight was significantly higher after 14 days of treatment compared to the first day of treatment for all groups. There was no significant difference between the groups in terms of body weight at the end of the treatment period (day 14) (Table 4). Daily food consumption was consistent throughout the study period and was not significantly different between groups (Table 4).

### 3.2. The Difference in Hepatic Nuclear Nrf2 Translocation Induced by Equal Doses of Different Dietary Chemicals

**Figure 1:**
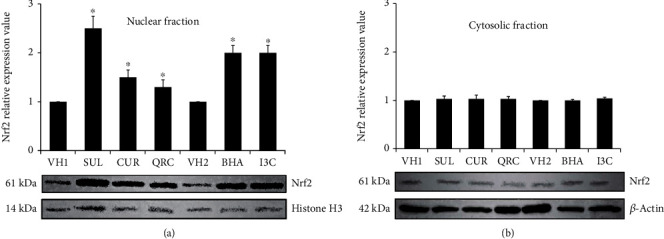
Administration of equal doses (50 mg/kg b.w.) of SUL, CUR, QRC, BHA, and I3C to mice for 14 days induces Nrf2 nuclear translocation in the liver *with different potencies*. (a) Nuclear fractions (100 *μ*g) from each mouse were separated by electrophoresis, and the expressions of proteins were analyzed by Western blotting. Nrf2 is the protein of interest, while histone H3 is the housekeeping protein for the nuclear fraction. The bands for Nrf2 and histone H3 were densitometrically scanned, and the expression of nuclear Nrf2 (relative to histone) was determined as fold increase of the vehicle-treated (control) animals as shown in the bar chart above. (b) Cytoplasmic fractions (100 *μ*g) from each mouse were separated by electrophoresis, and the expressions of proteins were analyzed by Western blotting. Nrf2 is the protein of interest, while *β*-actin is the housekeeping protein for the cytoplasmic fraction. The bands for Nrf2 and *β*-actin were densitometrically scanned, and the expression of Nrf2 in the nuclear fraction (relative to *β*-actin) was determined as fold increase of the vehicle-treated (control) animals as shown in the bar chart above. Values are expressed as the mean ± SEM (*n* = 4). Asterisk (∗) indicates a statistically significant difference from all control groups (vehicle 1 control and vehicle 2 control groups) after analysis using the Mann–Whitney test (*P* < 0.05). SUL: sulforaphane-treated group; CUR: curcumin-treated group; QRC: quercetin-treated group; BHA: butylated hydroxyanisole-treated group; I3C: indole 3 carbinol-treated group; VH1: control group treated with vehicle 1; VH2: control group treated with vehicle 2.

Treatment of mice with an equal dose (50 mg/kg) of dietary chemicals (SUL, BHA, I3C, CUR, and QRC) for 14 days significantly increased Nrf2 band intensity levels in the nuclear fraction of the liver compared to mice in the control groups (Figure 1(a)). This was complemented by low Nrf2 band intensity levels in the cytoplasmic fraction (Figure 1(b)), indicating that Nrf2 has translocated into the nuclear fraction. Quantitative analysis of the band intensity levels obtained for Nrf2 in the nuclear fraction, as optimized to that of histone H3 bands, revealed that SUL, BHA, I3C, CUR, and QRC treatment produced a significant induction of Nrf2 protein expression in the nuclear fraction, compared to the control groups (2.5-, 2.0-, 2.0-, 1.5-, and 1.3-fold, respectively, as compared to controls; *P* < 0.05) (Figure 1). In the cytoplasmic fraction, the Nrf2 bands of the treatment groups looked very faint, suggesting that Nrf2 has translocated into the nuclear fraction. Quantitative analysis of the band intensity levels obtained for Nrf2 in the cytoplasmic fraction, as optimized to that of *β*-actin bands, showed that the expression levels of Nrf2 in the SUL, BHA, I3C, CUR, and QRC groups are similar to the control groups (1.0-fold for all groups). The Nrf2 expression levels are also not significantly different from the control groups (Figure 1). The ratios of Nrf2 expression between the nuclear and cytoplasmic fractions for SUL, BHA, I3C, CUR, and QRC are 2.5-, 2.0-, 2.0-, 1.5-, and 1.3, respectively. Therefore, the potency of the dietary chemicals in inducing Nrf2 translocation was in the order of SUL > BHA = I3C > CUR > QRC. The results suggested that treatment with dietary chemicals (SUL, BHA, I3C, CUR, and QRC) led to the translocation of Nrf2 protein from the cytosol to the nucleus in mouse liver with differing potencies (Figure 1).

### 3.3. Administration of SUL, BHA, I3C, CUR, and QRC Did Not Affect NF-*κ*B Nuclear Translocation

**Figure 2:**
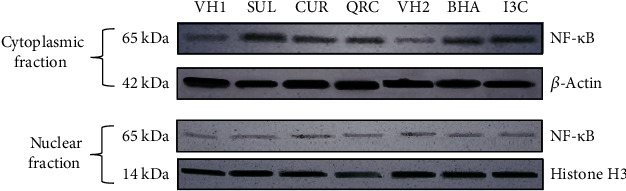
Administration of equal doses (50 mg/kg b.w.) of SUL, CUR, QRC, BHA, and I3C to mice for 14 days did not induce NF-*κ*B nuclear translocation.

To further investigate whether or not another oxidative stress-sensitive transcription factor apart from Nrf2 was activated by administration of different dietary chemicals, Western blot assays for the detection of NF-*κ*B expression were performed in the nuclear and cytoplasmic fractions derived from liver homogenates of mice treated with equal doses (50 mg/kg) of SUL, BHA, I3C, CUR, and QRC. Our results showed that there was no obvious NF-*κ*B induction or translocation in the liver nuclear fraction. The NF-*κ*B expression can only be prominently detected in the liver cytosolic fractions of mice in the control and treated groups, implying that the different dietary chemicals are not a trigger of NF-*κ*B activation in the liver (Figure 2).

### 3.4. The Different Patterns of Nrf2 Nuclear Translocation Induced by Different Dietary Chemicals Are Functionally Relevant In Vivo

**Figure 3:**
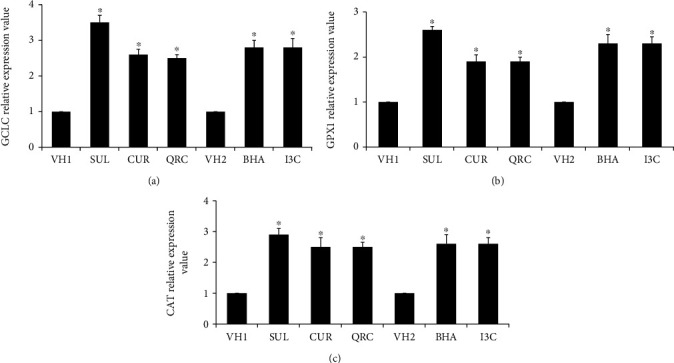
Effects of administration of 50 mg/kg SUL, CUR, QRC, BHA, and I3C for 14 days on (a) GCLC, (b) GPX1, and (c) CAT gene expressions in the livers of mice using RT-qPCR. GAPDH was designated as the reference gene. Data were represented as the mean ± SEM of six experiments, *n* = 6. Asterisk (∗) indicates a statistically significant difference compared to all control groups (vehicle 1 control and vehicle 2 control groups) after analysis using the Mann–Whitney test (*P* < 0.05). SUL: sulforaphane-treated group; CUR: curcumin-treated group; QRC: quercetin-treated group; BHA: butylated hydroxyanisole-treated group; I3C: indole 3 carbinol-treated group; VH1: control group treated with vehicle 1; VH2: control group treated with vehicle 2.

GCLC, GPX1, and CAT have been known to be regulated by Nrf2 [13, 57]. In this study, the effects of administration of equal doses of several dietary chemicals on GCLC, GPX1, and CAT gene expression were determined. As shown in Figures 3(a), 3(b), and 3(c), administration of equal doses (50 mg/kg) of SUL, BHA, I3C, CUR, and QRC to mice resulted in a significant increase in the fold change of liver gene expression levels of GCLC (3.5-, 2.8-, 2.8-, 2.6-, and 2.5-fold, respectively, as compared to controls; *P* < 0.05), GPX1 (2.6-, 2.3-, 2.3-, 1.9-, and 1.9-fold, respectively, as compared to controls; *P* < 0.05), and CAT (2.9-, 2.6-, 2.6-, 2.5-, and 2.5-fold, respectively, as compared to controls; *P* < 0.05). The GCLC, GPX1, and CAT-inducing potency of the chemicals was therefore in the order of SUL > BHA = I3C > CUR > QRC. Therefore, the order of relative expression level for each of the three genes (GCLC, GPX1, and CAT) follows the order of relative change of nuclear fraction of their transcription factor Nrf2. Thus, the different patterns of expression of Nrf2-regulated genes seen in the liver, even though the same dose of SUL, BHA, I3C, CUR, and QRC was administered to the animals, provided additional evidence that the dietary chemicals have different potencies in inducing stabilization and translocation of Nrf2 protein into the nuclear fraction.

## 4. Discussion

The effect of administration of equal doses (50 mg/kg) of SUL, BHA, I3C, CUR, and QRC to mice on liver Nrf2 nuclear translocation is the main objective of this study. In this study, healthy mice were used as the experimental model in order to mimic the situation whereby healthy humans are taking dietary supplements to find out whether there are any beneficial health effects seen in this case. If the extent of Nrf2 nuclear translocation is significantly increased and the gene expression of antioxidant proteins is significantly enhanced after administration of dietary chemicals in healthy mice, it strongly suggests that the dietary chemicals have the property of being chemoprotective if the mice encounter any disease or exposed to environmental and oxidative stress, which could be extrapolated back to humans. Subsequently, it was found that administration of equal doses (50 mg/kg) of SUL, CUR, QRC, BHA, and I3C to mice significantly induced the translocation of Nrf2 into the nuclear fraction of the liver with differing intensities. The differing intensities of Nrf2 translocation subsequently resulted in the different levels of expression of Nrf2-regulated genes (GCLC, GPX1, and CAT), but with a similar pattern as the Nrf2 nuclear translocation intensities (i.e., SUL > BHA = I3C > CUR > QRC) (Figures 1 and 3). The similar relative intensity values (i.e., the value of 1) of Nrf2 expression in the cytoplasmic fraction between controls and treatment samples indicated that the low expression levels of Nrf2 in the cytoplasmic fraction are similar throughout for controls and samples, as indicated by the faint Nrf2 bands compared to *β*-actin bands (Figure 1).

Even though we administered SUL, CUR, QRC, BHA, and I3C through intraperitoneal administration, the metabolism of the dietary chemicals in the liver should occur after intraperitoneal administration of the chemicals and not only exclusively after oral consumption [58]. Intraperitoneal administration was performed to lessen the complications in the absorptions of the various dietary chemicals. Therefore, the results of our studies should be valid because the dietary chemicals are all on equal footing when it comes to absorption, in the sense that the complexities arising from the poor absorption of the chemicals due to gastrointestinal absorption after oral consumption are eliminated by giving intraperitoneal absorption. After being administered intraperitoneally, the chemicals will enter the mesenteric blood vessels in the peritoneum, which eventually goes to the portal vein of the liver, and in the liver, the various dietary chemicals will be metabolized to their active metabolites [58]. It is most likely that in the liver, BHA will be metabolized to tert-butylhydroquinone (TBHQ) and tert-butylquinone (TBQ), whereas I3C will be metabolized to 3,3′-diindolylmethane (DIM), for example.

Certain chemicals with prooxidant properties alter the redox state of target cells directly through the generation of moderate amounts of reactive oxygen species (ROS) which disrupts the affinity between Nrf2 and Keap1, subsequently resulting in Nrf2 nuclear translocation. With this regard, the capability of dietary chemicals to induce Nrf2-regulated gene expression correlates well with their prooxidant properties [10]. Such chemicals with prooxidant properties include tert-butylhydroquinone (TBHQ) and butylquinone (TBQ), which are phase I metabolites of BHA in the liver [32, 33]. These prooxidants oxidize Keap1 thiols and release Nrf2 from Keap1 binding. Nrf2 is then able to enter the nucleus and activates the expression of chemopreventive genes [59]. This suggests the important chemoprotective role of BHA against liver oxidative injury and liver cancer.

Sulforaphane most possibly activates Nrf2 via the formation of thionoacyl adducts with Keap1 to release Nrf2 [60]. Modification of the critical cysteine thiols (C38, C151, C368, C489, C77, C226, C319, and C434) of Keap1 resulted in Nrf2 being translocated to the nucleus and activates ARE-responsive genes [26]. Further detailed studies revealed that sulforaphane preferentially reacts with the thiol group of cysteine 151 on the surface of Keap1. Any mutation in cysteine 151 of Keap1 severely abolished the activity of sulforaphane [60]. The two Michael acceptor groups characterizing the curcumin can interact readily with cysteine sulfhydryl groups of Keap1 via Michael addition and change the conformation of Keap1-Nrf2 complex, thereby releasing Nrf2 and promoting its activity [61]. Curcumin had been shown to induce nuclear translocation of Nrf2 and upregulate the expression of cytoprotective enzymes in rats afflicted with acute liver injury, which implies its protective role in the chemoprevention of liver injury [61].

Quercetin promotes Nrf2 nuclear translocation, the binding activity of nuclear proteins to the ARE, and increased the Nrf2-mediated gene transcription [62, 63]. Quercetin upregulated Nrf2 translocation in rat intestinal epithelial IEC-6 cells. Quercetin administration increased Nrf2 nuclear translocation in broiler chickens (LPS-induced intestinal oxidative stress) and male Sprague-Dawley rats (cerebral ischemia-induced oxidative stress). The downstream activation of the Nrf2/ARE pathway upon Nrf2 nuclear translocation by quercetin has been demonstrated through the CAT gene overexpression (which is Nrf2-regulated) [62, 64]. Previous research has shown that quercetin increases the mRNA level of GCLC and stimulates the nuclear translocation of Nrf2 in neuronal cells [65]. There is also convincing evidence to show that quercetin can modestly increase Nrf2 nuclear translocation in human hepatocytes, possibly mediated by p38 and endoplasmic reticulum kinase [66]. These findings suggest that quercetin could play an important role in the chemoprotective actions against liver injury, disease, and malignancy. In the present study, I3C induced the translocation of Nrf2 into the nuclear fraction and increased the expression of several Nrf2-regulated genes in the mouse liver. Therefore, our result indicated that I3C possessed the ability to increase the expression of Nrf2-regulated genes. DIM (the major metabolite of I3C) induced Nrf2 transactivation and the expression of genes regulated by Nrf2 (*γ*GCS, NQO1, and HO-1) [67]. DIM (and I3C) is a weaker Nrf2 agonist than SFN [67]. This result is similar to the result obtained in our present study.

Some of the Nrf2-regulated genes (such as HO-1 and GPX1) have binding sites for the oxidative stress-sensitive transcription factor nuclear factor-kappa-B (NF-*κ*B) in their regulatory regions [43–45]. In this study, we investigated whether equal doses (50 mg/kg) of sulforaphane, curcumin, quercetin, BHA, and I3C induced the translocation of NF-*κ*B into the nuclear fraction of the mouse liver with a similar pattern and intensity. The results of our study showed that equal doses of the aforementioned dietary chemicals inhibited the NF-*κ*B translocation in the nuclear fraction (Figure 2).

Dietary glucosinolate derivatives inhibit NF-*κ*B-mediated processes in vitro and in vivo. Glucosinolate derivatives (e.g., sulforaphane) are capable of inhibiting NF-*κ*B-regulated pathways by blocking proinflammatory signals at various levels; however, the precise molecular mechanisms by which these interactions are exerted are still not clearly understood. Previous studies suggested that sulforaphane suppressed IKK/IkB phosphorylation, inhibited IĸB and p65 NF-*κ*B nuclear translocation, and downregulated the transcriptional activity of NF-*κ*B, thus affecting the function of inflammatory mediators such as IL-6, iNOS, TNF-*α*, and COX-2 [24, 25, 68].

From the results of our study, it could be seen that curcumin was unable to induce NF-*κ*B translocation into the nuclear fraction (Figure 2). This is in agreement with the results from previous studies which showed curcumin convincingly inhibited NF-*κ*B activity in several cell types [69, 70]. The molecular mechanisms involved in the suppressive effects of flavonoids on NF-*κ*B are currently unknown, but several possibilities have been postulated [71]. The flavonoid quercetin is one such agent and has been found to possess potent anti-inflammatory effects, due in part to its anti-NF-*κ*B activity [72].

Our results indicated that butylated hydroxyanisole (BHA) caused inhibition of NF-*κ*B protein (Figure 2). Our results agreed with previous findings showing that BHA and its metabolite TBHQ suppressed NF-*κ*B activation [73, 74]. Our present study indicates that I3C inhibited NF-*κ*B protein expression. I3C and its metabolite DIM have been found to inhibit both inducible and constitutive NF-*κ*B activation, suppressed the activation of IKK, and prevented the nuclear translocation of p65 NF-*κ*B and NF-*κ*B-dependent gene expression in myeloid and leukemia cells [75–77].

Since NF-*κ*B nuclear translocation was inhibited in this study, it is therefore likely that the increased expression of phase II and antioxidant genes of interest in this study (GCLC, GPX1, and catalase) was most likely mediated by activation of Nrf2 observed in the mice administered with structurally diverse dietary chemicals (sulforaphane, curcumin, quercetin, BHA, and I3C), as indicated by an increased nuclear concentration of Nrf2 with different patterns according to each chemical characteristic. Indeed, studies have suggested that chemopreventive agents capable of inhibiting NF-*κ*B translocation, especially those derived from dietary agents, hold great promise in the treatment and prevention of cancer [78].

Following the aforementioned discussion, it can be concluded that a panel of equal doses (50 mg/kg) of small molecules, mainly involving compounds belonging to different structural classes, e.g., *α*-*β* unsaturated carbonyls, isothiocyanates, flavonoid, and polyphenols, with differing oxidative or electrophilic properties, were found to activate Nrf2 at different potencies. The selection of small molecules was based on their electrophilic nature, as in the case of the *α*-*β* unsaturated carbonyl-containing compounds and isothiocyanates, or based on their potential chemical or enzymatic conversion to electrophiles, similar to that of the flavonoids and phenolic compounds (Table 1). Including other likely mechanisms such as phosphorylation of Nrf2, compounds in this study that were capable of activating Nrf2 are postulated to be those that can modify the Keap1 thiols or be converted to compounds capable of such modifications. The final result is the disruption of the Keap1-Nrf2 complex, most likely through interaction with the thiols present on Keap1. Further studies are needed to conclusively determine the detailed mechanisms of Nrf2 release from Keap1 binding. In our discussion above, we only postulated the possible mechanisms in which these dietary chemicals cause the release of Nrf2 from Keap1 binding. The verification of the postulated mechanisms in which the dietary chemicals react with Keap1 is beyond the scope of our study. Further studies are needed to conclusively determine the interaction mechanisms between the dietary chemicals and Keap1 that causes the release of Nrf2. However, our study is still novel in the sense that no previous study has measured the difference in the potency of different dietary chemicals in inducing Nrf2 translocation in the liver using in vivo mouse studies. Most other studies of similar nature have used either cells or cell lines [67].

Previous cellular-based and animal studies have indicated that SUL, CUR, QRC, I3C, and BHA are involved in the chemoprevention of liver malignancy [24, 34, 36–39]. Studies utilizing animal models and cell culture have indicated that SUL, CUR, QRC, I3C, and BHA are involved in the chemoprevention of liver malignancy [24, 34, 36–39]. SUL, CUR, QRC, I3C, and BHA have also been shown to activate the Nrf2 pathway in the livers of animals such as rats and mice [15–20]. However, no previous studies have been attempted to find which dietary chemical is the most potent in inducing Nrf2 translocation in the liver nuclear fraction in vivo, and therefore theoretically the most potent in the chemoprevention of liver malignancy. Our animal-based study has indeed shown that in the mouse liver, SUL is the most potent dietary chemical in inducing Nrf2 nuclear translocation, followed by BHA and I3C (equipotent), CUR, and QRC. Previously, SUL has been implicated in many cellular-based studies as one of the most potent naturally occurring inducers of phase II enzymes [67, 79], and this conclusion is further confirmed by our animal-based study. The electrophilic tuning ability of sulforaphane enables it to be the most efficient in enhancing Nrf2 activation compared to other dietary chemicals used in this current study [67]. However, it is still unclear whether the combination of SUL together with one or more of the other dietary chemicals (CUR, QRC, I3C, and BHA) would further increase Nrf2 activation and the induction of its downstream genes, thus providing superior cancer chemoprevention effect than SUL alone. Further studies are needed to confirm this possibility.

The different induction profiles of the downstream genes regulated by Nrf2 indicate the differences in the fundamental mechanism of actions of Nrf2, at least at the mRNA level. Some possible reasons for the variations could be (1) differences in the arrangements of the AREs existing in the promoters of Nrf2-regulated genes, (2) differences in the composition of the ARE binding complexes in terms of the identities and relative quantities of the proteins concerned, (3) the levels of expression of genes and proteins induced vary depending on the nature of chemicals that elicits the response, and (4) different chemicals might activate the MAPK/PKC pathways in different ways, all of which could affect Nrf2-ARE activation at various intensities or levels.

## 5. Conclusion

Our findings indicated equal doses (50 mg/kg) of SUL, CUR, QRC, BHA, and I3C induced liver Nrf2 nuclear translocation with different potencies. The results also showed that equal doses (50 mg/kg) of SUL, CUR, QRC, BHA, and I3C could elicit different patterns of Nrf2-dependent gene expression levels in the liver. Sulforaphane is the most potent Nrf2 inducer amongst the dietary chemicals studied. The diverse nature of Nrf2 activators implies that the activation of Nrf2 is perhaps due to the binding of electrophilic functional groups of the dietary chemicals to Keap1 thiols. Although Nrf2 activation is not the only mechanism of protection conferred by the dietary chemicals, it is perhaps one of the most important mechanisms involved. Therefore, increased dietary consumption of vegetables (e.g., broccoli, cabbages, and kale), fruits (e.g., apples, grapes, and berries), and spices (e.g., onions and turmeric) is potentially the most practical way to induce Nrf2 activation in the human body and to provide chemoprevention against liver malignancy through induction of phase II enzymes in the liver. In the current study, we found that SUL is the most potent Nrf2 inducer, thus potentially and theoretically having the best cancer chemopreventive effect. However, the effect of the combination of SUL with other dietary chemicals has not been studied. It could be that the combination of SUL with one or more dietary chemicals would further enhance its cancer chemoprevention potency. Further studies are needed to confirm this possibility. Despite promising results in laboratory settings, the applicability of chemoprevention to human for any cancer has met with limited success largely due to inefficient systemic delivery and bioavailability of promising chemopreventive agents. We envision that nanoparticle-mediated delivery could be useful to limit the perceived toxicity and to enhance the bioavailability of the chemopreventive agents, and the concept of “nanochemoprevention” could be the way forward, where nanotechnology is incorporated for the enhancement of chemopreventive efficacy of agents. Neutraceutical-based supplements containing the dietary compounds used in this study, but formulated in such a way so as to have good oral absorption, should be the way forward for liver cancer chemoprevention. Clinical trials using nanoformulated potent Nrf2 activators in combination with standard treatments should be the focus of future research in liver cancer chemoprevention and treatment.

## Figures and Tables

**Table 1:** Dietary chemicals used in this study.

Dietary chemicals	Structure	Functional group	Source
Sulforaphane (SUL)	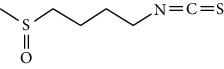	Isothiocyanate	Broccoli 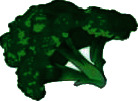
Curcumin (CUR)	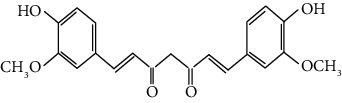	*α*,*β*-Unsaturated carbonyl (good Michael acceptor), 1,2-diphenol	Turmeric 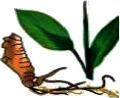
Quercetin (QRC)	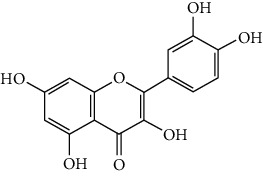	B ring *o*-dihydroxyl groups, the 4-oxo group in conjugation with the 2,3-alkene, and the 3-hydroxyl group	Onions, apples 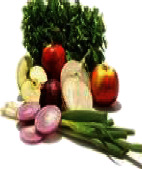
Butylated hydroxyanisole (BHA)	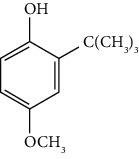	Methoxyphenol	Preservative in fats, foods, and cosmetics 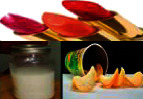
Indole-3-carbinol (I3C)	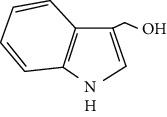	Indoles	Cabbage family 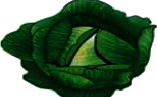

**Table 2:** Composition of standard pelletized diet (Gold Coin, Malaysia).

Composition	Amount/percentage
Crude protein	20%
Crude fibre (max)	5.0%
Crude fat (min)	2.5%
Moisture (max)	13.0%
Calcium	0.7-1.4%
Phosphorus	0.6–1.0%
Nitrogen-free extract	49.0%
Vitamin A	10 M.I.U
Vitamin D_3_	2.5 M.I.U
Vitamin E	15 g
Vitamin B_1_, vitamin B_2_, vitamin B_3_, vitamin B_5_, vitamin B_6_, vitamin B_12_, vitamin K, choline, Santoquin, microminerals	Trace amounts

**Table 3:** Oligonucleotide sequences used for real-time PCR reactions.

Gene	Primer sequences	Reference
GADPH	F: 5′-GTGGAGTCTACTGGTGTCTTCA-3′ R: 5′-TTGCTGACAATCTTGAGTGAGT-3′	Kong et al. [53]
GCLC	F: 5′-GCACGGCATCCTCCAGTTCCT-3′ R: 5′-TCGGATGGTTGGGGTTTGTCC-3′	Leung et al. [54]
CAT	F: 5′-GTGCGGACATTCTACACAAAGG-3′ R: 5′-GAACATTGCCGGCCACC-3′	Lu et al. [55]
GPX1	F: 5′-AGTCCACCGTGTATGCCTTCT-3′ R: 5′-GAGACGCGCGACATTCTCAATGA-3′	Nakamura et al. [56]

F: forward primer; R: reverse primer.

**Table 4:** Body weight and food intake of control and treated mice.

Groups	Body weight		Daily food intake (g/b.w./day)
	Day 1 (g)	Day 14 (g)	
VH1	26.25 ± 0.48	29.72 ± 0.52^∗^	4.27 ± 1.18
VH2	26.60 ± 0.50	29.75 ± 0.43^∗^	4.33 ± 0.16
SUL	27.23 ± 0.73	30.03 ± 0.65^∗^	4.62 ± 1.20
CUR	26.32 ± 0.28	29.50 ± 0.34^∗^	4.25 ± 1.38
QRC	26.55 ± 0.27	29.55 ± 0.33^∗^	4.71 ± 0.19
BHA	27.53 ± 0.71	30.63 ± 0.57^∗^	4.56 ± 1.93
I3C	26.47 ± 0.48	29.55 ± 0.47^∗^	4.53 ± 0.03

Values are given as the mean ± SEM (*n* = 6 mice in each group). Asterisk (∗) denotes significant difference in body weight at day 1 vs. day 14 (Mann–Whitney test; *P* < 0.05). SUL: sulforaphane-treated group; CUR: curcumin; QRC: quercetin; BHA: butylated hydroxyanisole; I3C: indole-3-carbinol; VH1: control group treated with vehicle 1; VH2: control group treated with vehicle 2.

## Data Availability

All data are available from the corresponding author under reasonable request.
